# Radiologic and endoscopic treatment for a disconnected pancreatic duct syndrome associated with percutaneous pancreatic fistula: achieving the “internalization of the fistula”

**DOI:** 10.1055/a-2088-8921

**Published:** 2023-06-12

**Authors:** Ilaria Tarantino, Lucio Carrozza, Giacomo Emanuele Maria Rizzo, Salvatore Tammaro, Luigi Maruzzelli, Dario Ligresti, Mario Traina

**Affiliations:** 1 Endoscopy Unit, Department of Diagnostic and Therapeutic Services, IRCCS – ISMETT Palermo, Italy; 2 Department of Surgical, Oncological and Oral Sciences (Di.Chir.On.S.), University of Palermo, Palermo, Italy; 3 Radiology Unit, Department of Diagnostic and Therapeutic Services, IRCCS – ISMETT, Palermo, Italy

A 33-year-old man was referred to our institution for the management of a percutaneous pancreatic fistula after acute pancreatitis due to SARS-CoV-2 infection. He developed a peripancreatic collection (PPC), which was percutaneously drained because of infection. After resolution of the collection, percutaneous leakage from the main pancreatic duct (MPD) was observed. The patient underwent endoscopic retrograde cholangiopancreatography (ERCP) with biliary and pancreatic sphincterotomy, and placement of both pancreatic and biliary stents; however, the leak persisted.

He was then referred to our institution, where initial management included ERCP with placement of two transpapillary pancreatic stents and the removal of the percutaneous catheter; however, fluid continued to leak from the percutaneous fistula ( Fig. 1 ). A multidisciplinary board decided to perform a rendezvous procedure with interventional radiology to facilitate endoscopic ultrasound (EUS) transgastric drainage of the pancreatic area that was currently draining into the percutaneous fistula ( Video 1 ) 1 2 3 .

**Fig. 1:**
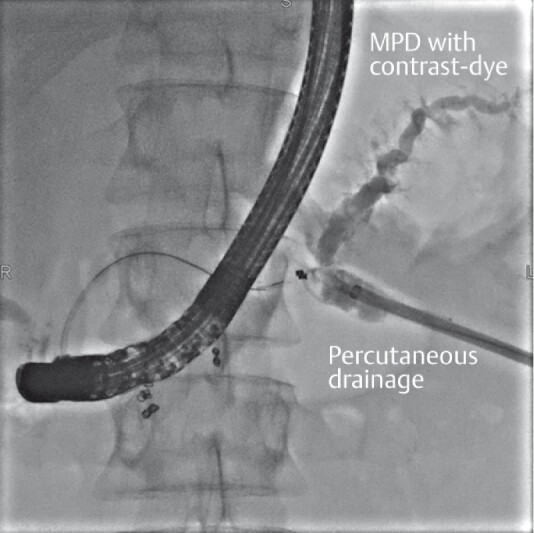
Fluoroscopic view of injection of contrast dye from the percutaneous fistula, showing no drainage into the duodenum through the two pancreatic stents.

**Video 1** Management of pancreato-cutaneous fistula after percutaneous drainage of peripancreatic collection.


The procedure included an initial ERCP with replacement of the two pancreatic stents ( Fig. 2 ) while the radiologist percutaneously placed a guidewire through the fistula to the point of leakage in the MPD. Then, using EUS, the point at which the percutaneous guidewire entered the MPD was identified and, under EUS guidance, a second guidewire was inserted transgastrically into the MPD through a 19-gauge needle and then advanced out through the percutaneous fistula ( Fig. 3 ). Dilation up to 10 mm was performed to create a transgastric pancreatic fistula.

**Fig. 2:**
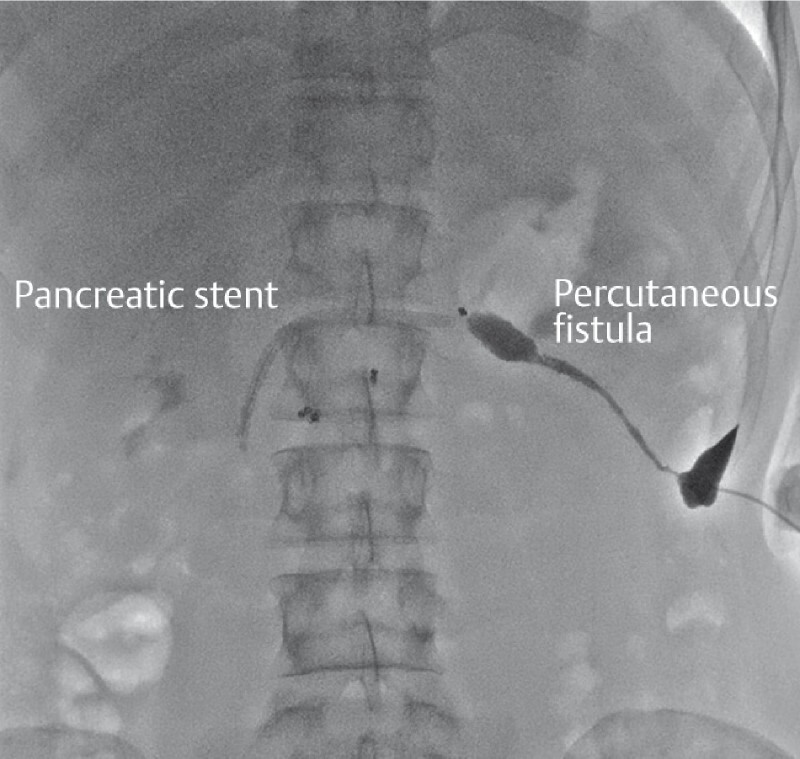
Fluoroscopic view through the percutaneous fistula showing normal aspect of pancreatography of the body and tail downstream of the interrupted main pancreatic duct.

**Fig. 3:**
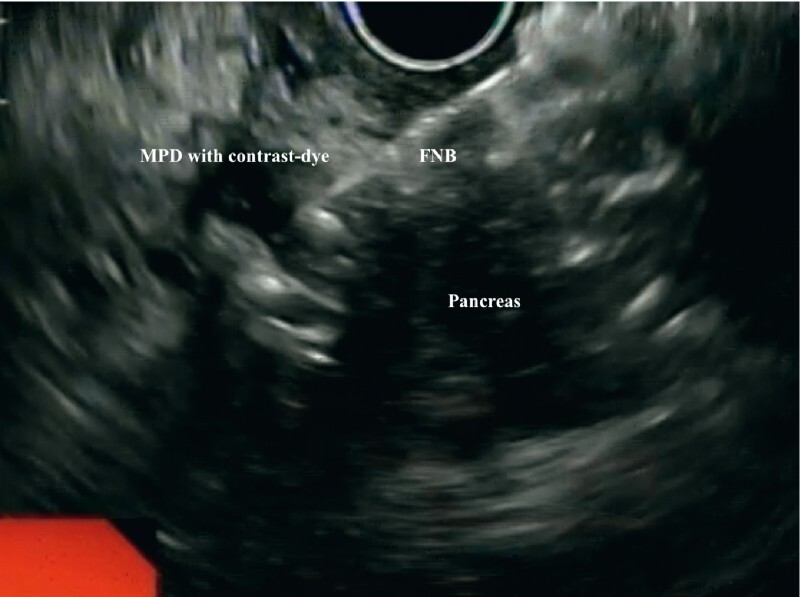
Endoscopic ultrasound (EUS) view of the transgastric EUS-guided insertion of a guidewire into the main pancreatic duct (MPD). FNB, fine-needle biopsy.

The next step was to percutaneously insert a double-pigtail (10 Fr) stent, releasing the distal side into the stomach and the proximal side into the MPD, in order to stabilize the newly created fistula. Another transgastric plastic stent was endoscopically placed through the gastropancreatic fistula ( Fig. 4 ). Injection of contrast dye through the percutaneous fistula confirmed complete drainage into the stomach.

**Fig. 4:**
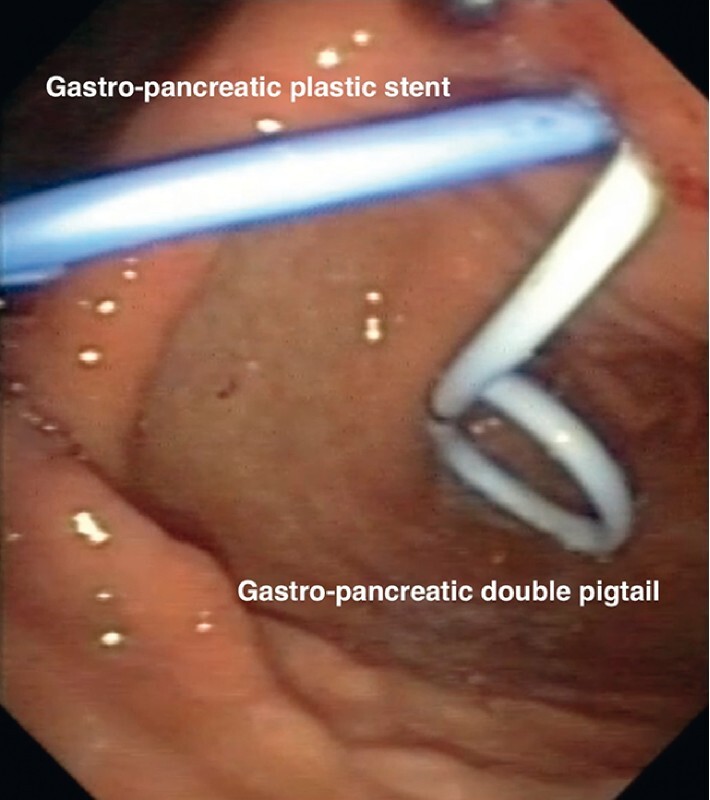
Double-pigtail stent and plastic stent through the newly created gastropancreatic fistula.

In conclusion, the procedure achieved complete exclusion and resolution of the pancreatic-cutaneous fistula.

Endoscopy_UCTN_Code_TTT_1AR_2AI

## Notes

Endoscopy E-Videos

https://eref.thieme.de/e-videos
*E-Videos* is an open access online section of the journal *Endoscopy* , reporting on interesting cases and new techniques in gastroenterological endoscopy. All papers include a high-quality video and are published with a Creative Commons CC-BY license. Endoscopy E-Videos qualify for HINARI discounts and waivers and eligibility is automatically checked during the submission process. We grant 100% waivers to articles whose corresponding authors are based in Group A countries and 50% waivers to those who are based in Group B countries as classified by Research4Life (see: https://www.research4life.org/access/eligibility/ ). This section has its own submission website at https://mc.manuscriptcentral.com/e-videos
